# Supplementary material for: Dissimilar Conservation Pattern in Hepatitis C Virus Mutant Spectra, Consensus Sequences, and Data Banks
Source: J Clin Med. 2020 Oct 27;9(11):3450. doi: 10.3390/jcm9113450 (PMC7692060; doi:10.3390/jcm9113450)
Supplement: Supplementary file 1 [file jcm-09-03450-s001.pdf]

Supplementary material for:

Dissimilar conservation pattern in hepatitis C virus mutant spectra, consensus sequences, and data banks

Carlos García-Crespo[1], María Eugenia Soria[1,2], Isabel Gallego[1,3], Ana Isabel de Ávila[1], Brenda Martínez-González[1,2], Lucía Vázquez-Sirvent[1], Jordi Gómez[3,4], Carlos Briones[3,5], Josep Gregori[3,6,7], Josep Quer[3,6], Celia Perales[1,2,3]*, Esteban Domingo[1,3]*

[1]Department of Interactions with the environment, Centro de Biología Molecular "Severo Ochoa" (CSIC-UAM), Consejo Superior de Investigaciones Científicas (CSIC), Campus de Cantoblanco, 28049, Madrid, Spain, [2]Department of Clinical Microbiology, IIS-Fundación Jiménez Díaz, UAM. Av. Reyes Católicos 2, 28040 Madrid, Spain, [3]Centro de Investigación Biomédica en Red de Enfermedades Hepáticas y Digestivas (CIBERehd) del Instituto de Salud Carlos III, 28029, Madrid, Spain, [4]Department of Molecular Biology, Instituto de Parasitología y Biomedicina 'López-Neyra' (CSIC), Parque Tecnológico Ciencias de la Salud, Armilla, 18016, Granada, Spain, [5]Department of Molecular Evolution, Centro de Astrobiología (CAB, CSIC-INTA), 28850 Torrejón de Ardoz, Madrid, Spain, [6]Liver Unit, Internal Medicine Hospital Universitari Vall d'Hebron, Vall d'Hebron Institut de Recerca (VHIR), 08035, Barcelona, Spain, [7]Roche Diagnostics, S.L., Sant Cugat del Vallés, 08174, Barcelona, Spain.

*Corresponding authors: Esteban Domingo (edomingo@cbm.csic.es) and Celia Perales (cperales@cbm.csic.es; celia.perales@quironsalud.es)

Table S1. Mutations and corresponding amino acid substitutions of the NS5A-NS5B-coding region in the mutant spectra of viral populations analyzed by ultra deep sequencing[a].

Mutation[b]	Amino Acid substitution[c]
A7650G[d]	**D461G**
U7651C[d]	Syn
U7651G[d]	**D461E**
A7652G[d]	**T462A**
A7652G[d]<br>C7653G[d]<br>C7654A[d]	**T462G**
A7652C[d]<br>C7653G[d]<br>C7954G[d]	**T462R**
A7655G[d]	**T463A**
A7655U[d]	**T463S**
C7656A[d]	**T463N**
C7656G[d]	**T463S**
G7658A[d]	**V464M**
G7658C[d]	**V464L**
G7658U[d]	**V464L**
U7659A[d]	**V464E**
U7661A[d]	**C465S**
U7661C[d]	**C465R**
C7681U	Syn
U7693G	Syn
A7696G	Syn
A7717G	Syn
U7727C	Syn
U7741C	Syn
G7744A	Syn
A7762C	Syn
U7768C	Syn
U7783C	Syn
A7786G	Syn
A7789G	Syn
U7802C	**S46P**
G7810A	Syn
U7825A	Syn
U7825C	Syn
U7842C	**V59A**
U7858C	Syn
A7864G	Syn
U7868C	Syn
A7870G	Syn
C7876U	Syn
G7886A	**A74T**
C7900G	Syn

C7903A	**S79R**
A7906C	Syn
A7906G	Syn
A7907U<br>G7908U	**R81L**
U7911G	**L82R**
C7915G	Syn
C7918U	Syn
U7919C	Syn
G7924A	Syn
G7930U	Syn
U7937C	Syn
U7942C	Syn
U7951C	Syn
U7954A	Syn
U7954C	Syn
A7957G	Syn
A7958G	**R98G**
A7965G	**K100R**
U7969C	Syn
A7972G	Syn
C7975U	Syn
G7978A	Syn
U7997C	Syn
U8000G	**S112A**
G8007A	**R114K**
C8020U	Syn
A8036G	**K124E**
U8068C	Syn
C8071U	Syn
A8074G	Syn
A8089G	Syn
U8092C	Syn
C8101U	Syn
G8107C	Syn
G8108A	**D148N**
G8114A	**A150T**
A8118G	**K151R**
U8125C	Syn
C8132G	**P156A**
C8132U	**P156S**
U8137C	Syn
A8144C	**I160L**
C8161U	Syn
G8162A	**G166S**
C8191U	Syn
U8200C	Syn
A8201G	**T179A**
G8209A	Syn
G8209U	**K181N**
U8215C	Syn
G8222A	**V186I**
U8233C	Syn

U8239C	Syn
C8242U	Syn
C8251U	Syn
C8260U	Syn
A8263G	Syn
U8275C	Syn
C8278G	Syn
C8278U	Syn
A8295G	**E210G**
U8310C	**M215T**
U8314G	Syn
U8317C	Syn
U8323C	Syn
U8326C	Syn
U8353C	Syn
G8374A	Syn
A8376G	**E237G**
C8386U	Syn
U8396C	**S244P**
C8398U	Syn
C8399U	Syn
C8404G	Syn
U8419C	Syn
C8421U	**A252V**
C8421U<br>C8422U	**A252V**
C8422U	Syn
A8427G	**H254R**
U8446G	Syn
A8455G	Syn
C8470U	Syn
A8475G	**K270R**
U8479C	Syn
A8483G	**T273A**
U8491C	Syn
U8491G	Syn
C8494U	Syn
G8518A	Syn
A8521U	Syn
C8530U	Syn
U8536C	Syn
C8539U	Syn
C8551U	Syn
A8566G	Syn
C8575U	Syn
A8577G	**K304R**
U8581C	Syn
G8584A	Syn
C8595A	**A310E**
A8602G	Syn
U8620C	Syn
A8626G	Syn

**Total Mutations[e]**	**145**
**Synonymous[f]**	**101**
**Non-synonymous[g]**	**44**

[a] The HCV populations analysed and the location of the mutations, encoded amino acid substitutions and their tolerability were previously described in Gallego et al, J Virol 94(6), 2020 doi: 10.1128/JVI.01856-19.

[b] The HCV genome residue numbering corresponds to the JFH-1 genome (accession number #AB047639); genomic residues 7649 to 8653 were analysed.

[c] Amino acid residues (single letter code) are numbered for the C-terminal part of NS5A and from the N- to the C-terminus of NS5B.

[d] Mutations (and deduced amino acid substitutions) of NS5A.

[e] Number of different mutations found counted relative to the sequence of the parental plasmid Jc1FLAG2(p7-nsGluc2A).

[f] Number of different synonymous substitutions found counted relative to the sequence of the parental plasmid Jc1FLAG2(p7-nsGluc2A).

[g] Number of different non-synonymous substitutions found counted relative to the sequence of the parental plasmid Jc1FLAG2(p7-nsGluc2A).

Table S2. Reference accession numbers of sequences retrieved from Los Alamos database.

Genotype 1	
**Subtype 1a**	AB520610[a], AF009606[a], AF011751 - AF011753[a], AF271632[a], AF511949[c], AF511950[c], AJ278830, EF407411 - EF407415, EF407417 - EF407419, EF407421 - EF407423, EF407425, EF407427, EF407428, EF407431 - EF407447, EF407449 - EF407457, EF621489, EU155214 - EU155216, EU155233, EU155236 - EU155245, EU155249 - EU155252, EU155265 - EU155278, EU155282 - EU155288, EU155291, EU155293, EU155294, EU155296, EU155297, EU155299, EU155309, EU155311, EU155313, EU155314, EU155319 - EU155323, EU155338 - EU155355, EU155378, EU155379, EU234064, EU234065, EU239715, EU239716, EU250017, EU255927 - EU255958, EU255963 - EU255971, EU255973 - EU255992, EU255994 - EU255999, EU256002 - EU256024, EU256026, EU256028 - EU256034, EU256036 - EU256044, EU256047 - EU256053, EU256055 - EU256058, EU256060, EU256067, EU256068, EU256070 - EU256074, EU256087, EU256094, EU256095, EU256097, EU256105 - EU256107, EU260396, EU362876, EU362877, EU362879, EU362880, EU362882, EU362884 - EU362887, EU362891 - EU362898, EU362901, EU482831, EU482832, EU482834 - EU482838, EU482840 - EU482848, EU482852 - EU482858, EU482861 - EU482873, EU482878, EU482882, EU482884, EU482887, EU482889, EU529676 - EU529681, EU569722, EU569723, EU595697 - EU595699, EU660383 - EU660385, EU660387, EU687193 - EU687195, EU781746 - EU781803, EU781805 - EU781822, EU862824, EU862827, EU862828, EU862830 - EU862832, EU862834, EU862839 - EU862841, FJ024087, FJ024274 - FJ024276, FJ024278, FJ024280 - FJ024282, FJ181999 - FJ182001, FJ205867 - FJ205869, FJ390394, FJ390395, FJ390399, FJ410172, GQ149768, JQ914271, JQ914272, JX463525 - JX463530, JX463532 - JX463538, JX463541 - JX463545, JX463551 - JX463615, JX463617 - JX463622, JX463624 - JX463626, JX463628 - JX463633, JX463635 - JX463638, KC844049, M62321[a], M67463, NC_004102[a].
**Subtype 1b**	AB049087 - AB049096, AB049098 - AB049101, AB080299[a], AB154177 - AB154206, AB191333, AB249644, AB426117[a], AB429050, AB435162[a], AB442219 - AB442222, AB691953, AB779562, AB779679, AF054247 - AF054259[a], AF165045 - AF165064, AF176573, AF207752 - AF207758, AF207760 - AF207774, AF208024, AF313916, AF333324[b], AF356827, AF483269, AJ000009, AJ132996[c], AJ132997, AJ238799, AJ238800, AY045702, AY587016[a], AY587844, D10750[b], D10934, D11168, D11355, D13558[b], D14484, D30613, D45172[a], D50480 - D50485, D63857, D85516, D89815, D89872[c], D90208, DQ071885, EF032892 - EF032894, EF407458 - EF407504, EU155217 - EU155232, EU155235, EU155253 - EU155264, EU155279 - EU155281, EU155300 - EU155308, EU155315 - EU155318, EU155324 - EU155337, EU155356 - EU155377, EU155381, EU155382, EU234061,

	EU234062, EU239714, EU255960 - EU255962, EU256000, EU256001, EU256045, EU256059, EU256061, EU256062, EU256064 - EU256066, EU256075 - EU256085, EU256088 - EU256092, EU256098 - EU256103, EU482833, EU482839, EU482849, EU482859, EU482860, EU482874, EU482875, EU482877, EU482879 - EU482881, EU482883, EU482885, EU482886, EU482888, EU529682, EU660386, EU660388, EU781825 - EU781832, EU862835, EU862837, FJ024086, FJ024277, FJ024279, FJ390396 - FJ390398, FJ478453, FN435993, GU133617 - GU451224, HQ110091, HQ639937, HQ639940, HQ639946, HQ639947, HQ719473, HQ912956 - HQ912959, JN120912, KC439481 - KC439527, KC844051, KC844052, L02836[c], M58335, M84754, M96362, U01214, U16362, U45476, X61596
**Subtype 1c**	AY051292[c], D14853, KC844047
**Genotype 2**	
**Subtype 2a**	AB047639 - AB047645, AB690460, AB690461[a], AF169002 - AF169005, AF177036[a], AF238481 - AF238485[c], AY746460[c], D00944[a], HQ639938, HQ639939, HQ639943 - HQ639945, JX014307[a], KC844043, KC967476, KF676351, KF676352, KF700370[a], NC_009823[c]
**Subtype 2b**	AB030907[a], AB559564, AB661373, AB661374, AB661376 - AB661378, AB661380 - AB661386, AB661389 - AB661393, AB661395 - AB661397, AB661399 - AB661403, AB661405 - AB661407, AB661409 - AB661422, AB661424 - AB661431, AF238486[c], AY232730 - AY232749, D10988, DQ430815, DQ430817, JQ745651[a], KC197226, KC844048, KC967477, KC967478
**Subtype 2c**	D50409, JX227950, JX227951, JX227965, JX227966, KC197227, KC197228, KC967479
**Subtype 2j**	HM777358, HM777359, JF735113, KC197232, KC197233
**Subtype 2k**	AB031663, JX227952, JX227953, KC197234
**Genotype 3**	
**Subtype 3a**	AB691595[a], AB691596[a], AB792683[a], AF046866[c], AY956467, D17763, D28917, DQ430819, DQ430820, DQ437509, GQ275355, GQ356200 - GQ356217, GU814263[c], HQ639941, HQ639942, HQ912953, JN714194, JQ717254 - JQ717260, KC844041, KF035123 - KF035127, NC_009824, X76918
**Genotype 4**	
**Subtype 4a**	AB795432, DQ418782 - DQ418784, DQ418787 - DQ418789, DQ988073 - DQ988079, GU814265[c], NC_009825, Y11604
**Subtype 4d**	DQ418786, DQ516083, EU392172, FJ462437, KC844045
**Subtype 4f**	EF589160, EF589161, EU392169, EU392170, EU392174, EU392175

**Infected patients**	**1136 (95.4%)**
**Clones**	**35 (2.9%)**
**Infected chimpanzees**	**3 (0.3%)**
**Undefined origin**	**17 (1.4%)**
**Total sequences**	**1191**

[a] Sequences corresponding to clones.

[b] Sequences corresponding to infected chimpanzee.

[c] Sequences of undefined origin.

Table S3. Mutations and corresponding amino acid substitutions deduced from the genomic sites with composition heterogeneity of the beginning of the NS2-coding region to the end of the NS5B-coding region in the genomic consensus sequence analysed by Sanger sequencing[a].

**Mutation[b]**	**Amino Acid substitution[c]**
U2838U/G	**F20F/C**
A2861A/G	**T28T/A**
U3001C/U	Syn
G3119A/G	**A114T/A**
C3271C/U	Syn
C3280C/U	Syn
U3550U/G	Syn
A3565A/U	Syn
U3674C/U	Syn
G3724A/G	Syn
A3802A/G	Syn
G3870A/G	**R147K/R**
C4099U/C	Syn
G4111G/A	Syn
C4159C/G	Syn
A4195G/A	Syn
G4216G/A	Syn
C4264G/C	Syn
A4286G/A	**I286V/I**
A4381G/A	Syn
A4402A/G	Syn
G4458A/G	**R343Q/R**
A4545A/C	**K372K/T**
U4591U/C	Syn
U4864U/C	Syn
G4954G/C	**E508E/D**
A4972A/G	Syn
U5200C/U	Syn
C5230U/C	Syn
G5378G/A	**A19A/T**
U5392U/C	Syn
A5396G/A	**I25V/I**
U5500U/C	Syn
C5506C/U	Syn
U5534U/C	Syn
A5551A/U	**Q22Q/H**
A5680G/A	Syn
A5683G/A	Syn
U5848U/C	Syn
A5954G/A	**I157V/I**

G6031G/A	Syn
G6126G/A	**R214R/K**
A6338G/A	**T24A/T**
U6350U/A	**F28F/I**
C6376U/C	Syn
C6412C/A	**A48A/D**
G6484G/U	**M72M/I**
A6491G/A	**T75A/T**
U6532U/C	Syn
A6636A/G	**Q123Q/R**
A6658A/G	Syn
A6686A/C	**I140I/L**
A6711A/U	**E148E/V**
G6732G/U	**G155G/V**
U6733U/C	Syn
G6748A/G	Syn
C6968A/C	**L234I/L**
A7001A/G	**T245T/A**
C7009G/C	**S247R/S**
G7081U/G	**E271D/E**
C7083C/A	**S272S/Y**
U7107U/C	**L280L/P**
A7110A/C	**E281E/A**
A7175G/A	**S303G/S**
U7181U/C	**F305F/L**
A7207A/U	Syn
A7218G/A	**Y317C/Y**
U7238A/U	**S324T/S**
A7302A/G	**K345K/R**
A7325G/A	**R353G/R**
G7345G/C	Syn
G7425A/G	**G386D/G**
C7444C/U	Syn
C7444C/A	Syn
A7452A/G	**E395E/G**
G7475G/A	**G403G/S**
A7494A/G	**E409E/G**
A7498A/G	Syn
G7499A/G	**G411S/G**
U7610U/C[d]	**S448S/P**
G7618G/A[d]	Syn
G7649G/A[e]	**D461D/N**
A7652G/A[e]	**T462A/T**
A7655U/A[e]	**T463S/T**
G7658A/G[e]	**V464M/V**
U7661A/U[e]	**C465S/C**
A7814A/G[e]	**K50K/E**
U7842U/C[e]	**V59V/A**

G7897G/A[e]	Syn
U7942C/U[e]	Syn
C7953C/G[e]	**S96S/C**
A7982A/G[e]	**K106K/E**
C8054C/G[e]	**P130P/A**
C8071C/U[e]	Syn
C8132U/C[e]	**P156S/P**
A8201A/G[e]	**T179T/A**
G8209U/G[e]	**K181N/K**
G8222G/A[e]	**V186V/I**
G8227G/A[e]	**M187M/I**
U8310C/U[e]	**M215T/M**
U8353C/U[e]	Syn
C8422U/C[e]	Syn
A8427A/G[e]	**H254H/R**
U8446G/U[e]	Syn
A8475A/G[e]	**K270K/R**
C8575C/U[e]	Syn
U8722A/U	**D352E/D**
A8752G/A	Syn
A8758G/A	Syn
C8854C/A	Syn
G8893G/A	Syn
U8929C/U	Syn
U9014G/U	**S450A/S**
C9385G/C	Syn
**Total Mutations[f]**	**114**
**Synonymous[g]**	**53**
**Non-synonymous[h]**	**61**

[a] The HCV populations analysed, the location of the mutations, and examples of sites with two nucleotides were previously described in Gallego et al, J Virol 94(6), 2020 doi: 10.1128/JVI.01856-19.

[b] The HCV genome residue numbering corresponds to the JFH-1 genome (accession number #AB047639); genomic residues 2780 to 9442 were analysed.

[c] Amino acid residues (single letter code) are numbered from N- to the C-terminus of each region; Syn means synonymous (no amino acid replacement).

[d] Residues located in the insertion found in the NS5A region of genotype 2.

[e] Residues were mutational waves were also analysed.

[f] Number of different mutations found counted relative to the sequence of the parental plasmid Jc1FLAG2(p7-nsGluc2A).

[g] Number of different synonymous substitutions found counted relative to the sequence of the parental plasmid Jc1FLAG2(p7-nsGluc2A).

[h] Number of different non-synonymous substitutions found counted relative to the sequence of the parental plasmid Jc1FLAG2(p7-nsGluc2A).

Table S4. Number of patients infected by each HCV subtype.

Genotype	Subtype	Number of patients
G1	**1a**	50
	**1b**	87
	**1l**	1
G2	**2c**	1
	**2i**	2
	**2j**	1
G3	**3a**	47
G4	**4d**	27
Mixed infections	**4d (69.3%) + 1b (30.6%)**	1
	**4a (98.5%) + 1b (1.5%)**	1
	**1b (80.1%) + 1a (19.9%)**	1
	**4d (96.7%) + 3a (3.3%)**	1
	**TOTAL**	**220**

Table S5. Location within NS5B amino acids 124 to 320 of the positions where amino acid substitutions were identified in infected patients[a].

Amino acid position[b]	Amino acid change	Number of variants[c]
**124[d]**	Yes	10
**125**	No	-
**126**	Yes	2
**127**	Yes	3
**128**	Yes	4
**129**	Yes	2
**130**	Yes	7
**131**	Yes	9
**132**	Yes	1
**133**	No	-
**134**	Yes	2
**135**	Yes	5
**136**	Yes	2
**137**	Yes	1
**138**	Yes	1
**139**	Yes	2
**140**	No	-
**141**	Yes	1
**142**	Yes	3
**143**	Yes	3
**144**	Yes	1
**145**	Yes	3
**146**	Yes	3
**147**	Yes	4
**148[d]**	Yes	6
**149**	Yes	1
**150[d]**	Yes	7
**151[d]**	Yes	3
**152**	No	-
**153**	Yes	1
**154**	Yes	2
**155**	Yes	2
**156[d]**	Yes	3
**157**	Yes	1
**158**	Yes	1
**159**	Yes	1
**160**	Yes	2
**161**	Yes	1
**162**	Yes	4
**163**	No	-

**164**	Yes	1
**165**	No	-
**166[d]**	Yes	1
**167**	Yes	3
**168**	Yes	1
**169**	Yes	4
**170**	Yes	1
**171**	Yes	7
**172**	Yes	2
**173**	Yes	4
**174**	Yes	2
**175**	Yes	2
**176**	Yes	2
**177**	Yes	4
**178**	Yes	4
**179[d]**	Yes	4
**180**	Yes	7
**181[d]**	Yes	6
**182**	Yes	3
**183**	Yes	2
**184**	Yes	11
**185**	Yes	4
**186**	Yes	3
**187**	Yes	3
**188**	Yes	2
**189**	Yes	10
**190**	Yes	4
**191**	Yes	1
**192**	Yes	1
**193**	Yes	2
**194**	Yes	1
**195**	Yes	2
**196**	Yes	3
**197**	Yes	1
**198**	Yes	5
**199**	Yes	1
**200**	No	-
**201**	Yes	1
**202**	Yes	3
**203**	Yes	2
**204**	Yes	1
**205**	Yes	3
**206**	Yes	10

**207**	Yes	2
**208**	Yes	1
**209**	Yes	5
**210**[d]	Yes	8
**211**	Yes	2
**212**	Yes	3
**213**	Yes	8
**214**	No	-
**215**[d]	Yes	4
**216**	Yes	1
**217**	Yes	2
**218**	Yes	3
**219**	Yes	2
**220**	Yes	2
**221**	Yes	1
**222**	Yes	2
**223**	Yes	1
**224**	Yes	2
**225**	Yes	1
**226**	No	-
**227**	Yes	1
**228**	Yes	2
**229**	Yes	1
**230**	Yes	2
**231**	Yes	6
**232**	Yes	1
**233**	Yes	2
**234**	No	-
**235**	Yes	6
**236**	Yes	3
**237**[d]	Yes	1
**238**	Yes	3
**239**	Yes	3
**240**	Yes	2
**241**	Yes	1
**242**	Yes	2
**243**	No	-
**244**	Yes	5
**245**	Yes	4
**246**	Yes	5
**247**	Yes	2
**248**	Yes	3
**249**	Yes	2
**250**	Yes	3

**251**	Yes	5
**252[d]**	Yes	4
**253**	Yes	3
**254[d]**	Yes	7
**255**	Yes	3
**256**	Yes	1
**257**	Yes	2
**258**	Yes	4
**259**	Yes	2
**260**	Yes	1
**261**	Yes	1
**262**	Yes	4
**263**	Yes	1
**264**	No	-
**265**	Yes	1
**266**	Yes	2
**267**	Yes	5
**268**	Yes	1
**269**	No	-
**270[d]**	Yes	4
**271**	Yes	2
**272**	Yes	4
**273**	Yes	5
**274**	No	-
**275**	No	-
**276**	Yes	4
**277**	Yes	1
**278**	Yes	3
**279**	Yes	2
**280**	No	-
**281**	No	-
**282**	Yes	2
**283**	Yes	1
**284**	No	-
**285**	Yes	5
**286**	Yes	3
**287**	Yes	1
**288**	Yes	2
**289**	Yes	2
**290**	Yes	1
**291**	Yes	2
**292**	No	-
**293**	Yes	3

**294**	Yes	1
**295**	Yes	1
**296**	Yes	1
**297**	Yes	4
**298**	Yes	1
**299**	Yes	1
**300**	Yes	11
**301**	Yes	1
**302**	Yes	1
**303**	Yes	3
**304[d]**	Yes	4
**305**	Yes	2
**306**	Yes	2
**307**	Yes	7
**308**	Yes	2
**309**	Yes	4
**310[d]**	Yes	6
**311**	Yes	5
**312**	Yes	6
**313**	Yes	5
**314**	Yes	3
**315**	Yes	1
**316**	Yes	4
**317**	No	-
**318**	Yes	2
**319**	Yes	1
**320**	Yes	1
**Total of positions with amino acid changes**		**177**
**Total of positions without amino acid changes**		**20**
**Total of variants**		**522**

[a] The clinical history of the 220 patients cohort under study was described in Chen et al., Antiviral Research 174: 104694, 2020.

[b] The HCV genome residue numbering corresponds to the H77 genome (accession number #AF009606); genomic residues 7971 to 8561 were analysed.

[c] Number of different amino acid changes detected in each position relative to the respective reference sequence. Multiple amino acid substitutions per site are explained by the different HCV genotypes and subtypes of the sequence under study (Chen et al., Antiviral Research 174: 104694, 2020).

[d] Position where a variant amino acid is common in an infected patients and some cell culture mutant spectrum.

Table S6. Random distribution of positions in the HCV genome.

Randomized positions[a]		
**Control 1[b]**	**Control 2[b]**	**Control 3[b]**
2819	2799	2781
2906	2820	2840
2942	2866	2896
3093	2900	3048
3133	2904	3085
3220	2933	3407
3233	2961	3442
3272	3112	3603
3279	3146	3626
3360	3274	3628
3367	3361	3669
3453	3362	3690
3471	3399	3693
3493	3421	3694
3648	3467	3703
3657	3483	3733
3667	3705	3754
3669	3768	3837
3697	3839	3842
3882	3907	3916
4095	4034	3970
4103	4128	4008
4304	4133	4103
4332	4140	4149
4489	4158	4184
4517	4233	4190
4551	4303	4194
4761	4304	4202
4772	4305	4212
4812	4340	4455
4830	4362	4459
4998	4385	4471
5143	4397	4489
5155	4418	4568
5223	4432	4700
5246	4434	4784
5247	4537	4795
5296	4543	4918
5324	4551	4996
5393	4696	5058
5394	4711	5126

5493	4747	5157
5519	4766	5264
5745	4814	5323
5750	5168	5414
5799	5288	5485
5912	5335	5492
5994	5456	5535
6051	5528	5610
6054	5551	5687
6220	5569	5851
6394	5572	5878
6512	5603	5913
6597	5695	6028
6630	5769	6078
6647	5821	6231
6693	5851	6256
6705	5937	6388
6764	6019	6427
6765	6046	6522
6777	6131	6542
6794	6149	6562
6826	6171	6593
6879	6173	6601
6890	6203	6616
6910	6244	6625
6934	6270	6776
6959	6282	6788
6969	6388	6803
7054	6389	6955
7201	6443	7024
7266	6771	7069
7267	6805	7085
7426	6818	7132
7438	6825	7145
7613	6837	7207
7677	6904	7247
7726	6923	7283
7806	6956	7569
7810	7025	7575
7948	7062	7638
7950	7076	7671
8003	7090	7783
8064	7131	7861
8109	7139	7897
8126	7145	7966
8148	7187	8049

8156	7207	8060
8164	7286	8064
8195	7370	8131
8309	7397	8145
8357	7514	8385
8379	7577	8402
8390	7584	8420
8562	7733	8569
8622	7983	8602
8667	8002	8632
8690	8168	8675
8701	8180	8695
8711	8194	8709
8740	8304	8721
8750	8468	8736
8751	8505	8760
8789	8663	8772
8797	8707	8808
8815	8827	8881
8836	8830	8920
8868	8926	9038
8883	8927	9047
8939	8933	9178
9226	9021	9195
9233	9106	9279
9335	9117	9362
9337	9292	9378

[a]The HCV genome residue numbering corresponds to the JFH-1 genome (accession number #AB047639); genomic residues 2780 to 9442 were analysed.

[b] The assignments were as directed by Excel 2016.

Table S7. Statistical analysis of the different distributions identified between HCV genomic residues 2769 (beginning of the NS2-coding region) and 9377 (end of the NS5B-coding region) (H77 numbering).

Comparison	Panel[a]	p-value<br>Chi-square test Monte Carlo correction	Significance[b]
Distribution of heterogeneity sites relative to most abundant nucleotide	3A	0.0165	*
Random position distribution relative to the most abundant nucleotide	S2I		
Distribution of heterogeneity sites relative to Jc1Luc plasmid	3B	0.0045	**
Random position distribution relative to Jc1Luc plasmid	S2J		
Distribution of positions from Los Alamos alignment relative to the most abundant nucleotide	S2A	0.9725	ns
Random position distribution relative to the most abundant nucleotide	S2I		
Distribution of positions from Los Alamos alignment relative to Jc1Luc plasmid	S2B	1.0000	ns
Random position distribution relative to Jc1Luc plasmid	S2J		

[a] Panel means figure number and panel in main text or supplemental material (S).

[b] The statistical significance of the differences is given as follows: ns, not significant; *, $P \leq 0.05$; **, $P < 0.01$.

Figure S1

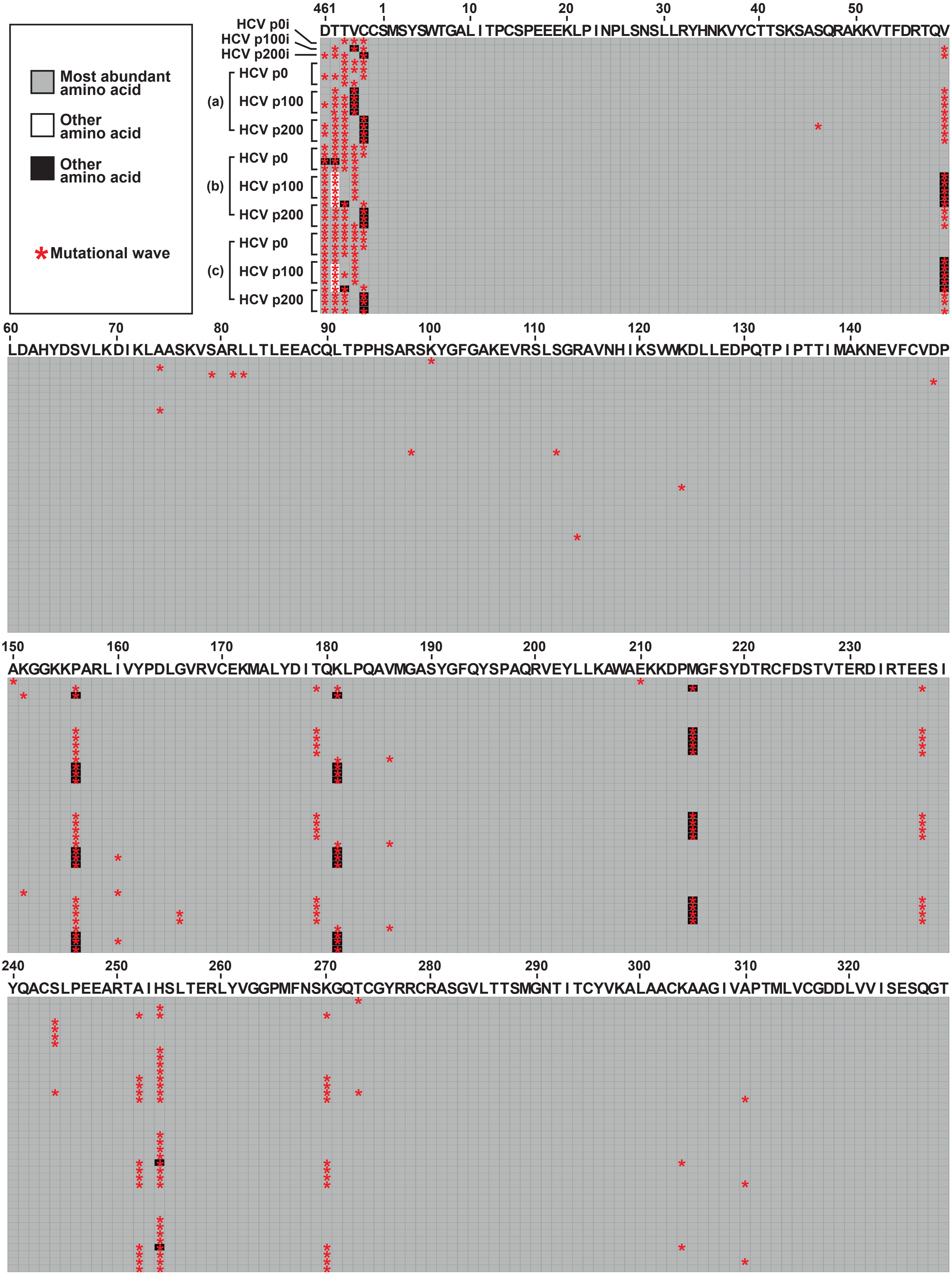
Most abundant amino acid
Other amino acid
Other amino acid
Mutational wave
HCV p0i
HCV p100i
HCV p200i
(a)
(b)
(c)
HCV p0
HCV p100
HCV p200
461 1 10 20 30 40 50
DTTVCCSMSYSWTGALITPCSPEEEKLPINPLSNSLLRYHNKVYCTTSKSASQRAKKVTFDRTQV
60 70 80 90 100 110 120 130 140
LDAHYDSVLKDIKLAASKVSARLLTLEEACQLTPPHSARSKYGFGAKEVRSLSGRAVNHIKSVWKDLLEDPQTPIPTTIMAKNEVFCVDP
150 160 170 180 190 200 210 220 230
AKGGKKPARLIVYPDLGVRVCEKMALYDITQKLPQAVMGASYGFQYSPAQRVEYLLKAWAEKKDPMGFSYDTRCFDSTVTERDIRTEESI
240 250 260 270 280 290 300 310 320
YQACSLPEEARTAIHSLTERLYVGGPMFNSKGQTCGYRRCRASGVLTTSMGNTITCYVKALAACKAAGIVAPTMLVCGDDLVVISESQGT

Figure S1. Heat map of an alignment of the 39 consensus amino acid sequences determined for the NS5A-NS5B region [encoded by nucleotides 7649 to 8653; numbering according to isolate JFH-1 (GenBank accession number #AB047639). The map for amino acids spans the same region for nucleotides shown in Fig. 2 of the main text. Each horizontal line represents the sequence of a population, as written on the left. The three first lines correspond to the initial populations, and the blocs below them include the populations at passages 1 to 4 of replicas (a), (b) and (c) of the three viral populations. Each column represents one of the 335 amino acid positions analyzed. Amino acid numbers for each protein are written above the amino acid sequence, which displays the most represented amino acid at each position in the sequences under comparison. The upper left box indicates the code for amino acid abundance: grey means an abundant amino acid (present in 66.7 % to 100 % of the compared sequences); white and black represent others amino acids presents in the same position. In NS5A a black square means the presence of amino acid E, R, S, M, and S at positions 461, 462, 463, 464, and 465, respectively, and white squares indicate the presence of amino acid A at position 462. In NS5B, black squares indicate the presence of amino acids A, S, N, T, and R, at positions 59, 156, 181, 215, and 254, respectively. Red asterisks point to amino acids encoded by nucleotides that participated in mutational waves (depicted in Fig. 2 of main text, and compiled in Table S1).

Figure S2

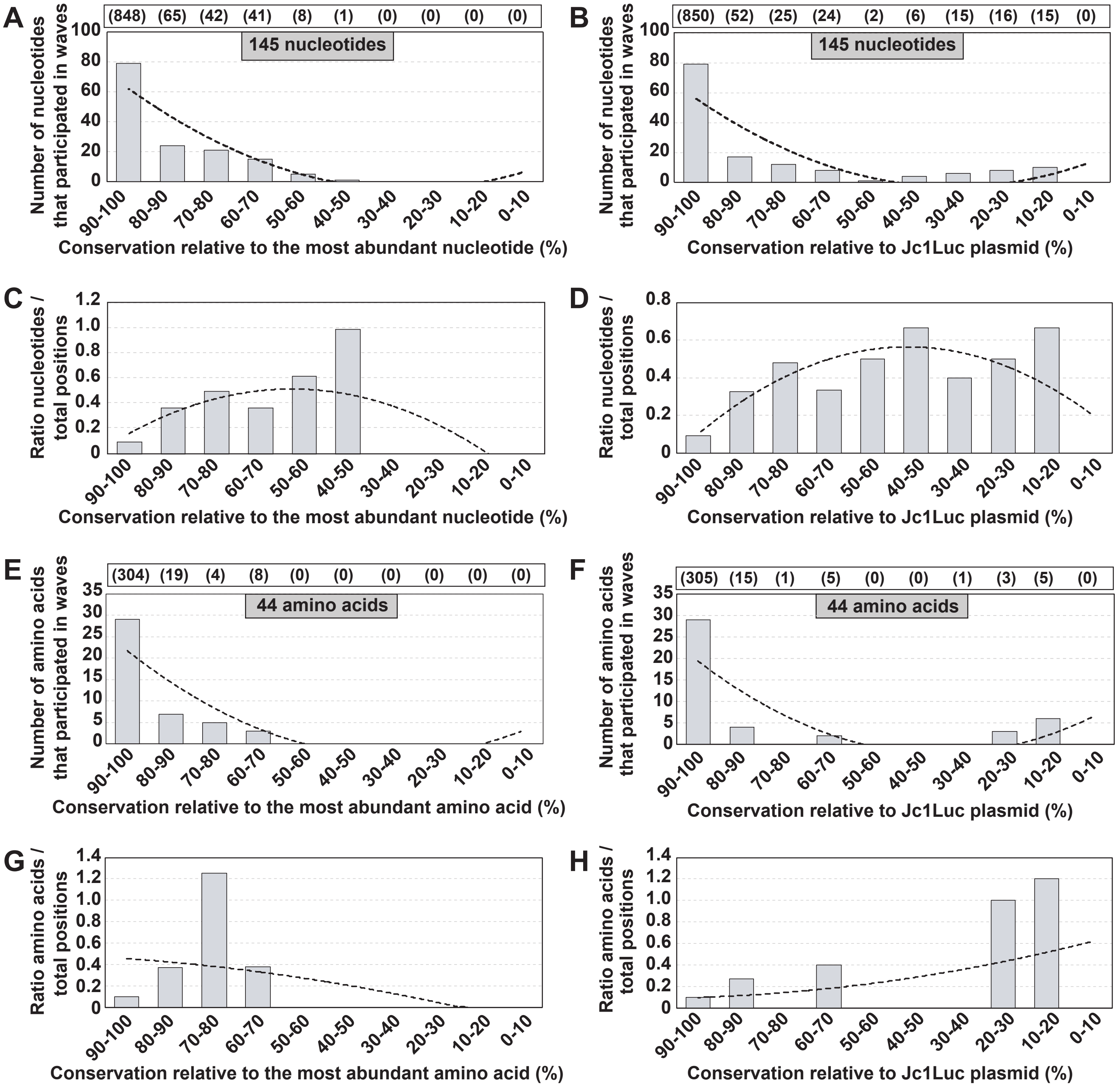
A
(848) (65) (42) (41) (8) (1) (0) (0) (0) (0)
145 nucleotides
Number of nucleotides that participated in waves
Conservation relative to the most abundant nucleotide (%)
B
(850) (52) (25) (24) (2) (6) (15) (16) (15) (0)
145 nucleotides
Number of nucleotides that participated in waves
Conservation relative to Jc1Luc plasmid (%)
C
Ratio nucleotides / total positions
Conservation relative to the most abundant nucleotide (%)
D
Ratio nucleotides / total positions
Conservation relative to Jc1Luc plasmid (%)
E
(304) (19) (4) (8) (0) (0) (0) (0) (0) (0)
44 amino acids
Number of amino acids that participated in waves
Conservation relative to the most abundant amino acid (%)
F
(305) (15) (1) (5) (0) (0) (1) (3) (5) (0)
44 amino acids
Number of amino acids that participated in waves
Conservation relative to Jc1Luc plasmid (%)
G
Ratio amino acids / total positions
Conservation relative to the most abundant amino acid (%)
H
Ratio amino acids / total positions
Conservation relative to Jc1Luc plasmid (%)
90-100 80-90 70-80 60-70 50-60 40-50 30-40 20-30 10-20 0-10

Figure S2. Degree of conservation of nucleotides and amino acids that participated in mutational waves, according to the LANL alignment using only sequences from genotype 2a (33 sequences). **(A)** Number of nucleotides involved in mutational waves distributed among conservation groups, calculated relative to the most abundant nucleotide at the corresponding position in the LANL alignment. Conservation groups are indicated in abscissa, and the number of nucleotides that participated in mutational waves in each group is given in ordinate. The total number of nucleotides within residues 7584 to 8588 (H77 numbering) from the alignment that fall in each conservation category is indicated in parenthesis in the upper box. The discontinuous line corresponds to function $y = 1.64x^2 – 24.26x – 84.93$ ($R^2 = 0.8596$). (B) Same as A but with nucleotide conservation in the LALN alignment calculated relative to the corresponding residues in plasmid Jc1Luc [28]. The discontinuous line corresponds to function $y = 1.69x^2 – 23.33 + 77.77$ ($R^2 = 0.6934$). (C) Data of A normalized to the number of residues in each conservation group; normalization was done by dividing the latter number by the total number of residues from the LANL alignment that fell into the corresponding group. The discontinuous line corresponds to function $y = -0.026x^2 + 0.24x – 0.05$ ($R^2 = 0.4623$). (D) Data of B normalized to the number of residues in each conservation group. The discontinuous line corresponds to function $y = -0.02x^2 + 0.23x – 0,11$ ($R^2 = 0.0.5205$). (E-H) Same as A-D but at the amino acid level. The defining functions are E: $y = 0.66x^2 – 9.33x + 30.37$ ($R^2 = 0.805$); F: $y = 0.7x^2 – 9.15x+ 27.75$ ($R^2 = 0.6049$); G: $y = -0.004x^2 + 0.021x + 0.48$ ($R^2 = 0.2571$); H: $y = 0.005x^2 + 0.003x 0.089$ ($R^2 = 0.1581$). The position of each mutation and amino acid substitution is given in Table S1, and control calculations and simulations of the mutant distributions are described in Figure S4.

Figure S3

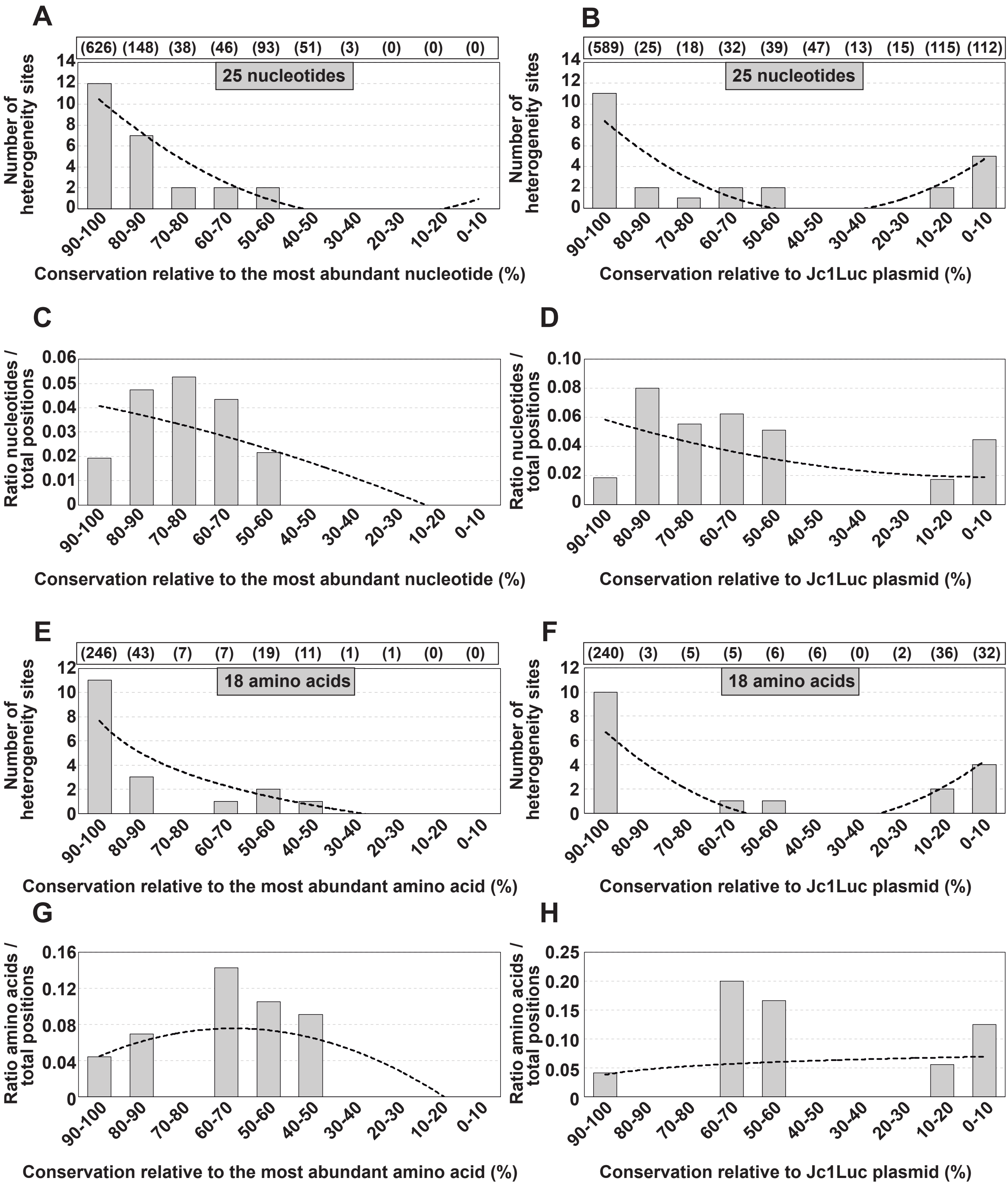
A
(626) (148) (38) (46) (93) (51) (3) (0) (0) (0)
25 nucleotides
Number of heterogeneity sites
Conservation relative to the most abundant nucleotide (%)
B
(589) (25) (18) (32) (39) (47) (13) (15) (115) (112)
25 nucleotides
Number of heterogeneity sites
Conservation relative to Jc1Luc plasmid (%)
C
Ratio nucleotides / total positions
Conservation relative to the most abundant nucleotide (%)
D
Ratio nucleotides / total positions
Conservation relative to Jc1Luc plasmid (%)
E
(246) (43) (7) (7) (19) (11) (1) (1) (0) (0)
18 amino acids
Number of heterogeneity sites
Conservation relative to the most abundant amino acid (%)
F
(240) (3) (5) (5) (6) (6) (0) (2) (36) (32)
18 amino acids
Number of heterogeneity sites
Conservation relative to Jc1Luc plasmid (%)
G
Ratio amino acids / total positions
Conservation relative to the most abundant amino acid (%)
H
Ratio amino acids / total positions
Conservation relative to Jc1Luc plasmid (%)
90-100
80-90
70-80
60-70
50-60
40-50
30-40
20-30
10-20
0-10

Figure S3. Number of nucleotides within HCV genomic residues 7584-8588 (H77 numbering) and deduced amino acid mixtures that belonged to heterogeneity sites, distributed among conservation groups according to the Los Alamos data base alignment. **(A)** Number of nucleotides at heterogeneity sites distributed among conservation groups, calculated relative to the most abundant nucleotide at the corresponding position in the Los Alamos alignment. The total number of nucleotides that fall in each conservation category is indicated in parenthesis in the upper box. The discontinuous line corresponds to function $y = 0.2652x^2 - 3.9773x + 14.167$ ($R^2 = 0.9117$). **(B)** Same as A but with nucleotide conservation in the Los Alamos alignment calculated relative to the corresponding residues in plasmid Jc1FLAG2(p7-nsGLuc2A (Marukian et al., Hepatology 48:1843-1850, 2008). The discontinuous line corresponds to function $y = 0.3371x^2 - 4.1144x + 12.15$ ($R^2 = 0.7324$). **(C)** Data of A normalized to the number of residues in each conservation group. The discontinuous line corresponds to function $y = -0.0002x^2 - 0.0031x + 0.0441$ ($R^2 = 0.5957$). **(D)** Data of B normalized to the number of residues in each conservation group. The discontinuous line corresponds to function $y = 0.0005x^2 - 0.0096x + 0.0676$ ($R^2 = 0.2183$). **(E-H)** Same as A-D but at the amino acid level. The defining functions are E: $y = -3.869\ln(x) + 7.6443$ ($R^2 = 0.6988$); F: $y = 0.3068x^2 - 3.6417x + 10.017$ ($R^2 = 0.6202$); G: $y = -0.0031x^2 + 0.0255x + 0.0227$ ($R^2 = 0.3955$); H: $y = 0.0136\ln(x) + 0.0383$ ($R^2 = 0.0168$). The position of each mutation and amino acid substitution deduced from the sites displaying composition heterogeneity is given in Table S3. The number of sequences retrieved from the Los Alamos data bank and inclusion criteria are explained in the main text.

Figure S4

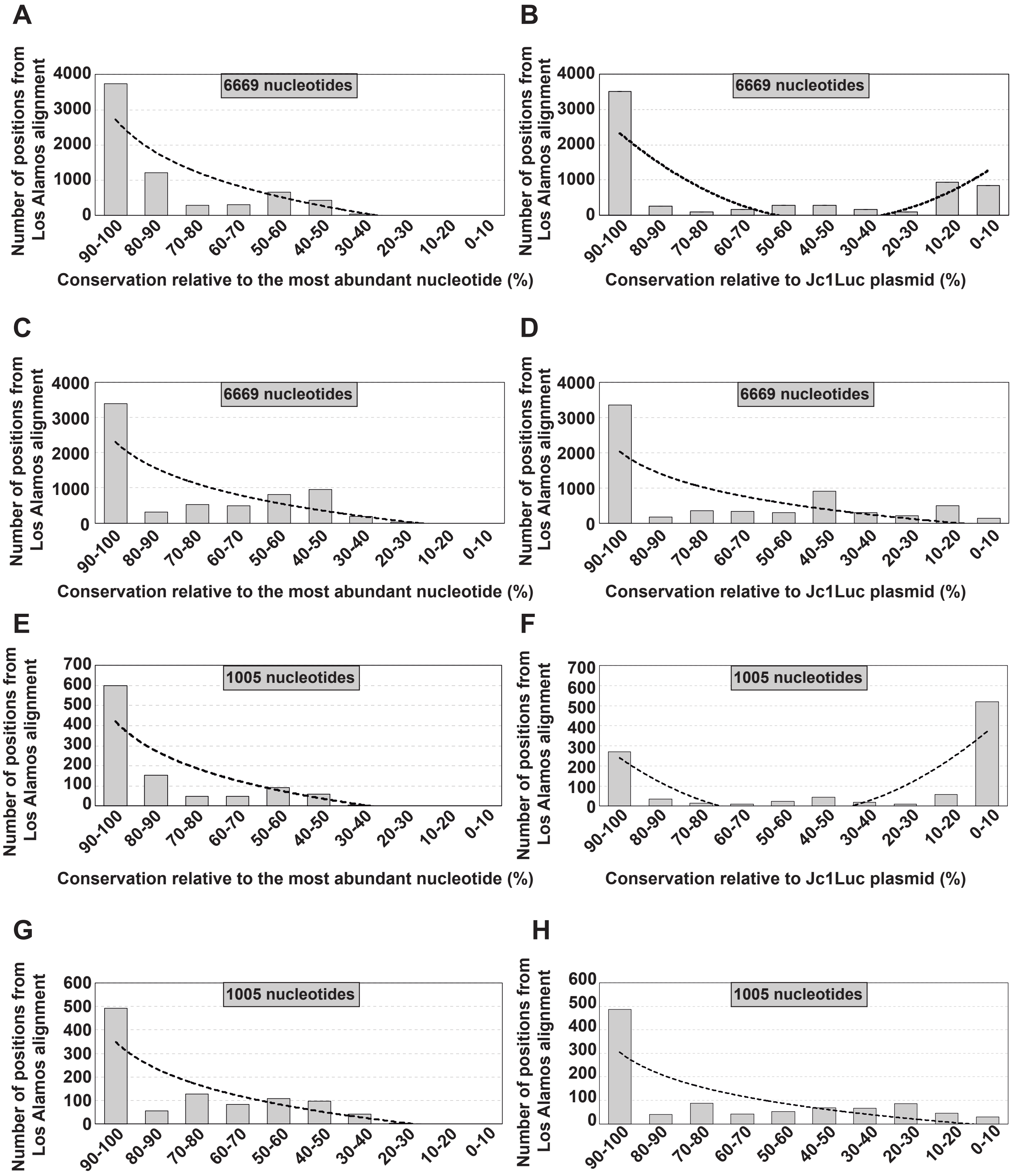
A
B
C
D
E
F
G
H
6669 nucleotides
1005 nucleotides
Number of positions from Los Alamos alignment
Conservation relative to the most abundant nucleotide (%)
Conservation relative to Jc1Luc plasmid (%)
90-100
80-90
70-80
60-70
50-60
40-50
30-40
20-30
10-20
0-10

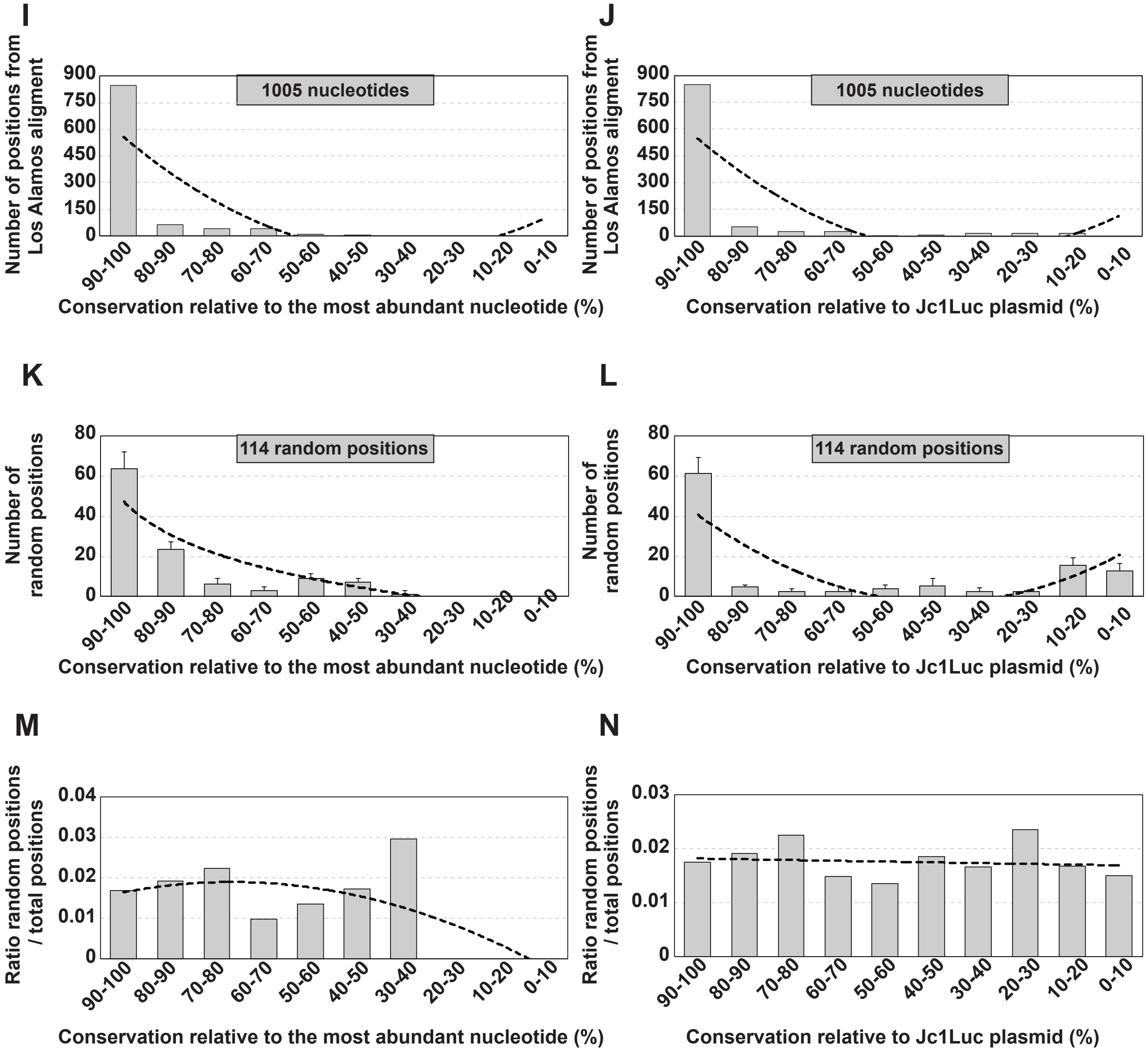
I
J
K
L
M
N
1005 nucleotides
1005 nucleotides
114 random positions
114 random positions
Number of positions from Los Alamos aligment
Number of random positions
Ratio random positions / total positions
900
750
600
450
300
150
0
80
60
40
20
0.04
0.03
0.02
0.01
0.03
90-100
80-90
70-80
60-70
50-60
40-50
30-40
20-30
10-20
0-10
Conservation relative to the most abundant nucleotide (%)
Conservation relative to Jc1Luc plasmid (%)

Figure S4. Resampling with specific genotypes, and simulations of distribution of HCV genomic nucleotides among conservation groups according to the LANL sequence alignment. **(A)** The positions comprised between the beginning of the NS2-coding region and the end of the NS5B-coding region (residues 2769 to 9377; H77 numbering) of an alignment of the HCV G1 and G2 genotypes (1112 sequences) were distributed in different windows according to the conservation of the most abundant nucleotide in each position of the Los Alamos alignment. Conservation groups are indicated in abscissa, and the number of positions that belongs to each group is indicated in ordinate. The discontinuous line corresponds to function $y = -1363\ln(x) + 2726.3$ ($R^2 = 0.7598$). **(B)** The positions were distributed in different windows according to the conservation of the nucleotide in the in the Los Alamos alignment, calculated using the HCV sequence in plasmid Jc1FLAG2(p7-nsGLuc2A) as reference. Conservation groups are indicated in abscissa, and the number of positions that belongs to each group is indicated in ordinate. The discontinuous line corresponds to function $y = 94.405x^2 - 1156x + 3390.2$ ($R^2 = 0.5997$). **(C, D)** Same as A and B except that conservation in the Los Alamos alignment was calculated using the same number of sequences of G1 and G2 (129 sequences of each genotype). The defining functions are C: $y = -1079\ln(x) + 2297.3$ ($R^2 = 0.6065$); D: $y = -910.9\ln(x) + 2042.8$ ($R^2 = 0.4731$). **(E, F)** Same as A, B except that the residues distributed among conservation groups are those of the NS5A-NS5B-coding region (residues 7584 to 8588; H77 numbering) for G1, G2, G3 and G4 genotypes (1191 sequences). The defining functions are E: $y = -212.8\ln(x) + 421.91$ ($R^2 = 0.7329$); F: $y = 17.186x^2 – 174.47x + 398.42$ ($R^2 = 0.695$). **(G, H)** Same as E, F except that 28 sequences of each of genotypes G1, G2, G3 and G4 were used for the alignment. The defining functions are G: $y = -164.6\ln(x) + 349.15$ ($R^2 = 0.6864$); H: $y = -135\ln(x) + 304.37$ ($R^2 = 0.5198$). **(I, J)** Same as E, F, except that 33 sequences from genotype 2a were used for the alignment. The defining functions are I: $y = 19.004x^2 – 260.11x + 799.48$ ($R^2 = 0.6485$); J: $y = 19.106x^2 – 258.51x + 786.73$ ($R^2 = 0.6158$). **(K)** Triplicate simulation (using program Excel 2016) of the distribution of 114 random positions (within residues 2769 to 9377 (H77 numbering) (the same number of heterogeneity sites found in the HCV cell culture quasispecies) among conservation ranges calculated relative to the conservation of the most abundant nucleotide in the Los Alamos alignment. Conservation group are indicated in abscissa. The average and corresponding standard deviations of random positions is indicated in ordinate. The discontinuous line corresponds to function $y = -23.71\ln(x) + 47.219$ ($R^2 = 0.7806$). **(L)** Same as I except that conservation in the Los

Alamos alignment was calculated relative to the HCV sequence in plasmid Jc1FLAG2(p7-nsGLuc2A). The discontinuous line corresponds to function $y = 1.6187x^2 – 19.999x + 59.078$ ($R^2 = 0.601$). **(M)** Ratio of the average of random positions that belong to each range of conservation group (calculated relative to the most abundant nucleotide as in panel I to the number of nucleotide positions of each group. The discontinuous line corresponds to function $y = -0.0005x^2 + 0.0031x + 0.0138$ ($R^2 = 0.4824$). **(N)** Ratio of the average of random positions that belong to each range of conservation group (calculated relative to corresponding nucleotide in the reference sequence Jc1FLAG2(p7-nsGLuc2A) as in panel J to the total nucleotide positions of each group. The discontinuous line corresponds to function $y = 0.0183e^{-0.008x}$ ($R^2 = 0.0187$). The random positions used for these controls are given in Table S4.

References

1. Gallego I, et al. (2020) Broad and Dynamic Diversification of Infectious Hepatitis C Virus in a Cell Culture Environment. J Virol 94(6).

2. Chen Q, et al. (2020) Deep-sequencing reveals broad subtype-specific HCV resistance mutations associated with treatment failure. Antiviral Res 174:104694.
